# Thrombomodulin regulates monocye differentiation *via* PKCδ and ERK1/2 pathway *in vitro* and in atherosclerotic artery

**DOI:** 10.1038/srep38421

**Published:** 2016-12-02

**Authors:** Chien-Sung Tsai, Yi-Wen Lin, Chun-Yao Huang, Chun-Min Shih, Yi-Ting Tsai, Nai-Wen Tsao, Chin-Sheng Lin, Chun-Che Shih, Hellen Jeng, Feng-Yen Lin

**Affiliations:** 1 Division of Cardiovascular Surgery, Tri-Service General Hospital, National Defense Medical Center, Taipei, Taiwan; 2 Division of Cardiology and Cardiovascular Research Center, Taipei Medical University Hospital, Taipei, Taiwan; 3 Department and Graduate Institute of Pharmacology, National Defense Medical Center, Taipei, Taiwan; 4 Division of Cardiovascular Surgery, Department of Surgery, Taoyuan Armed Forces General Hospital, Taoyuan, Taiwan; 5 Institute of Oral Biology, National Yang-Ming University, Taipei, Taiwan; 6 Departments of Internal Medicine, College of Medicine, School of Medicine, Taipei Medical University, Taipei, Taiwan; 7 Division of Cardiovascular Surgery, Taipei Medical University Hospital, Taipei, Taiwan; 8 Division of Cardiology, Tri-Service General Hospital, National Defense Medical Center, Taipei, Taiwan; 9 Division of Cardiovascular Surgery, Taipei Veterans General Hospital, Taipei, Taiwan; 10 Division of Anatomy and Cell Biology, College of Medicine, School of Medicine, Taipei Medical University, Taipei, Taiwan

## Abstract

Thrombomodulin (TM) modulates the activation of protein C and coagulation. Additionally, TM regulates monocyte migration and inflammation. However, its role on monocyte differentiation is still unknown. We investigated the effects of TM on monocyte differentiation. First, we found that TM was increased when THP-1 cells were treated with phorbol-12-myristate-13-acetate (PMA). Overexpression of TM enhanced the macrophage markers, CD14 and CD68 expression in PMA-induced THP-1. TM siRNA depressed the PMA-induced increase of p21^Cip1/WAF1^ via ERK1/2-NF-kB p65 signaling. TM regulated cytoskeletal reorganization via its interaction with paxillin, cofilin, LIMK1, and PYK2. In addition, PMA-induced p21^Cip1/WAF1^ expression, CD14-positive cell labeling intensity and ERK1/2 phosphorylation were markedly inhibited when protein kinase C-δ (PKCδ) was knocked down. We identified that TM directly interacts with PKCδ. PKCδ was highly expressed in human atherosclerotic arteries and colocalized with TM in CD68-positive infiltrated macrophages of plaques, indicating that the coordination between TM and PKCδ in macrophages participated in atherogenesis. TM may act as a scaffold for PKCδ docking, which keeps PKCδ in the region close to the monocyte membrane to promote the activation of ERK1/2. Taken together, our findings suggest that TM-PKCδ interaction may contribute to cardiovascular disorders by affecting monocye differentiation, which may develop future therapeutic applications.

Monocytes undergo transendothelial migration and differentiate into macrophages^1^, which take on the morphology and functional properties that are characteristic of the tissue they enter^2^. Atherosclerosis is considered as both a chronic inflammatory disease and a lipid metabolism disorder in which macrophages are responsible for intracellular lipid accumulation as well as foam cell formation in early atherosclerotic lesions^3^,^4^,^5^. After the induction of hyperlipidemia and in response to chemotactic factors, monocytes exit the peripheral bloodstream and then enter the arterial intima, where they further differentiate into macrophages after exposure to environmental factors, such as oxidized low-density lipoprotein (oxLDL), *Chlamydia pneumoniae*, and macrophage growth factors^6^,^7^,^8^. In addition, macrophages contribute to atherosclerotic lesion development through the production and release of a variety of soluble mediators, such as chemokines, cytokines, growth factors, and reactive oxygen species^9^. Furthermore, macrophages also play roles in plaque disruption and vascular remodeling in atherosclerosis by releasing matrix-degrading proteinases as well as their activators and inhibitors^10^. A previous paper showed that atherosclerotic lesions are fewer and smaller in apoE-deficient mice that completely lack macrophage colony-stimulating factor (M-CSF), an important promoter of macrophage differentiation, compared to mice deficient in only apoE^11^, confirming that macrophage differentiation is a critical component of atherogenesis^9^. Hence, the prevention of macrophage differentiation could confer protection from atherosclerosis with multiple pathologic effects.

Human THP-1 monocytic cells can be induced to differentiate into macrophages by treatment with phorbol-12-myristate-13-acetate (PMA), after which they can be converted into foam cells by exposure to oxLDL^12^. Schwende *et al*. reported that the differentiation statuses of THP-1 cells that were induced by PMA and 1,25-dihydroxyvitamin D3 are different. PMA treatment, which activates protein kinase C (PKC), induces a greater degree of differentiation in THP-1 cells, as is reflected by their increased adherence and expression of macrophage differentiation-associated surface markers^13^. PKCs, a family of serine/threonine kinases, play important roles in several signal transduction cascades and are known to be involved in inflammation, cell growth, and migration^14^. The PKC family consists of fifteen isozymes in humans. In addition to its classic (α, βI, βII, γ) and novel (δ, ε, η, θ) members, the PKC family includes two atypical members (ξ, λ) that are distinguished structurally by the presence of only one PKC zinc finger module in their regulatory domain and biochemically by their inability to bind to and respond to phorbol esters and diacylglycerol^15^,^16^.

Thrombomodulin (TM), a glycosylated type I transmembrane protein^17^, was first discovered on endothelial cells, where the TM/thrombin complex has been shown to modulate the activation of protein C and the inactivation of coagulation^18^. In 2008, recombinant human soluble thrombomodulin (rTM) was approved for the treatment of disseminated intravascular coagulation in Japan. TM is also expressed on keratinocytes^19^, megakaryocytes, platelets^20^, and monocytes^21^, indicating that TM has additional functions in addition to its role as an anticoagulant. It has been hypothesized that TM also functions as an angiogenic factor^22^, an adhesion molecule^23^, and an anti-inflammatory agent^24^,^25^. Moreover, our previous study demonstrated that down-regulation of TM in monocytes is associated with TNF-α production and poor early outcomes in patients following serious surgery^26^. Knockdown of TM enhanced IL-6-induced migration and chemotaxis in monocytes, indicating that TM plays important roles in monocyte functions^27^. A previous study^28^ reported that exposure of THP-1 cells to recombinant human soluble thrombomodulin (rTM) induced growth arrest and differentiation in THP-1 cells by activating JNK/c-Jun signaling, suggesting that rTM is a positive regulator of THP-1 differentiation. Although it has been suggested that TM is a potent stimulator of monocyte migration and differentiation, the roles of endogenous TM in THP-1 differentiation and its regulating mechanisms are not yet fully understood. Thus, the aim of this study was to investigate the mechanism of action underlying the effects of TM on PMA-induced monocyte differentiation.

Here, we demonstrate that TM expression was increased during PMA-induced THP-1 cell differentiation. Furthermore, we suggested that the mechanisms underlying TM-regulated monocyte differentiations are likely to be mediated through a direct interaction with PKCδ, which is thought to induce ERK1/2 activation and to cause THP-1 cell differentiation. This study may provide a new view of the role of endogenous TM in macrophage differentiation in atherogenesis.

## Results

### Thrombomodulin was associated with PMA-induced monocytic THP-1 cell differentiation

**Figure 1:**
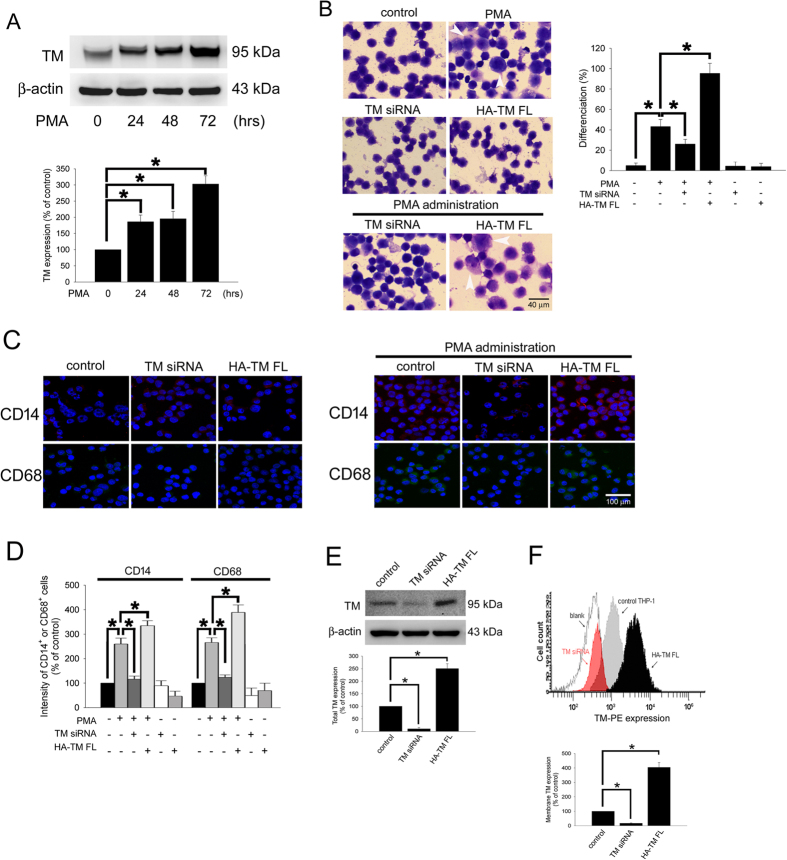
PMA-induced TM expression mediates morphological changes and differentiation marker expression in THP-1 cells. (**A**) THP-1 cells were treated with 150 nM PMA for 24–72 hours. The total cell lysates were harvested, and the expression of TM was analyzed using western blot analysis. β-actin was used as a loading control. Five independent experiments have been performed (n = 5) and representative images have been showed. The amount of proteins expression was quantified using densitometry and presented as bar graph. The data are presented as the mean ± SD (n = 5), and *p < 0.05 was considered significant. (**B**) THP-1 cells were transfected with TM siRNA or HA-TM FL plasmid for 24 h followed by PMA stimulation for 72 hours. The morphology of the cells was observed using light microscopy. The adherent differentiated macrophage-like cells are indicated by a white arrowhead. Five independent experiments have been performed (n = 5). The quantification is shown in the right graph. (**C**) The expression of the macrophage cell surface markers CD14 (red) and CD68 (green) was analyzed using immunofluorescence and microscopy. Hoechst staining was used to label the nuclei. The scale bar indicates 100 μm. Five independent experiments have been performed (n = 5), and showed representative images. (**D**) The expression of CD14 and CD68 was analyzed using flow cytometry. Data are expressed as a % of the control, are presented as the mean ± SD and represent the results of three independent experiments (n = 3, *p < 0.05 was considered significant). (**E**) THP-1 cells were transfected with TM siRNA or HA-TM FL plasmid for 24 h, the TM in total cell lysate was analyzed using western blot analysis. The amount of proteins expression was quantified using densitometry and presented as bar graph. (**F**) The membrane TM was stained by PE-conjugated anti-TM antibody and analyzed using flow cytometry. The relative amount of membrane TM expression was presented as bar graph. The data are presented as the mean ± SD (n = 5), and *p < 0.05 was considered significant.

TM is expressed on the surface of monocytes and is important for the regulation of THP-1 cell migration^27^. We wanted to determine whether TM is also involved in monocyte differentiation. We first determined the expression of TM during PMA-induced THP-1 cell differentiation into macrophages. The data indicated that treatment of THP-1 cells with 150 nM PMA for 24–72 h markedly increased the expression of TM in a time-dependent manner (Fig. 1A), suggesting that TM played a role in PMA-induced monocytic THP-1 cell differentiation. Cell adhesion and Liu staining were used to verify THP-1 cell differentiation. The quantitative data showed that treatment of THP-1 cells with PMA for 72 h significantly induced differentiation by 43.2 ± 6.9% (2160 ± 345 differentiated cells/per 5000 THP-1 cells) relative to the untreated control group (5.1 ± 2.5%; 255 ± 125 differentiated cells/per 5000 THP-1 cells; Fig. 1B). To verify that TM contributed to the effects of PMA-induced THP-1 cell differentiation, THP-1 cells were transfected with either TM siRNA for knockdown or HA-TM FL plasmid for overexpression for 24 h, which was followed by PMA treatment for 72 h. The results showed that knockdown of TM significantly decreased PMA-induced monocyte differentiation by 26.1 ± 4.5% (1305 ± 225 differentiated cells/per 5000 THP-1 cells). In contrast, overexpression of TM followed by PMA treatment significantly enhanced THP-1 cell differentiation by 95.4 ± 9.9% (4770 ± 495 differentiated cells/per 5000 THP-1 cells; Fig. 1B). The THP-1 cells were harvested, and the macrophage surface markers CD14 and CD68 were analyzed using immunostaining and flow cytometry. As shown in Fig. 1C,D, these cell surface antigens were strongly induced by 259.7 ± 24.3% for CD14 and 265.8 ± 19.4% for CD68 after the THP-1 cells were exposed to PMA to induce differentiation. siRNA knockdown of TM markedly decreased PMA-induced expression of CD14 by 116.4 ± 12.4% and CD68 by 123.9 ± 9.5% relative to the PMA-treated group. Conversely, the levels of CD14 and CD68 were significantly increased by 334.6 ± 20.1% and 388.9 ± 30.4%, respectively, when THP-1 cells were transfected with HA-TM FL plasmid followed by PMA stimulation. Hence, down-regulation of TM markedly reduced and up-regulation of TM greatly enhanced the macrophage-like phenotypes that were induced by PMA treatment. Additionally, transfection of THP-1 cells with TM siRNA or HA-TM FL plasmid alone did not increase the CD14 and CD68 expression. These data suggest that TM was involved in PMA-induced THP-1 cell differentiation. The flow cytometry and western blot analysis were used to define the efficiency of TM siRNA and HA-TM FL plasmid (Fig. 1E,F).

### TM modulates p21^Cip1/WAF1^ expression via ERK1/2 signaling, which reduces cell growth during PMA-induced THP-1 cell differentiation

**Figure 2:**
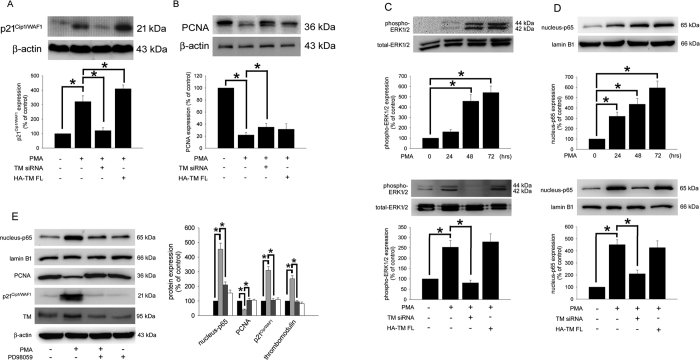
TM regulates PCNA and p21^Cip1/WAF1^ expression via ERK1/2 activation in PMA-stimulated THP-1 cells. (**A**,**B**) THP-1 cells were transfected with TM siRNA or HA-TM FL plasmid for 24 h, which was followed by PMA stimulation for 72 h. The expression of p21^Cip1/WAF1^ and PCNA in total cell lysates was assayed using western blot analysis. (**C**, upper and **D**, upper) THP-1 cells were stimulated with 150 nM PMA for 24–72 h.The expression of phosphorylated ERK1/2 in total cell lysates and NF-κB p65 in nuclear fraction were assayed using western blot analysis. (**C**, lower and **D**, lower), THP-1 cells were transfected with TM siRNA or HA-TM FL plasmid for 24 h, which was followed by PMA stimulation for 72 h. The expression of phosphorylated ERK1/2 and NF-κB p65 was assayed using western blot analysis. (**E**) THP-1 cells were pretreated with 10 μM PD98059 for 1 h followed by PMA treatment (dark gray) or treated with PD98059 only (white) for 72 h (black: naïve THP-1 cells; light gray: PMA treatment). A western blot assay was performed to determine the expression of NF-kB p65 (nuclear fraction), PCNA, and p21^Cip1/WAF1^ (total cell lysates). Lamin B1, total ERK1/2, and β-actin were used as the loading controls, as indicated. Five independent experiments have been performed (n = 5) for all studies, and representative images have been showed. The density of each band was quantified using densitometry and related protein expression was presented as bar graph. The data are presented as the mean ± SD, and *p < 0.05 was considered significant.

TM contributes to the effects of PMA-induced THP-1 cell differentiation; the molecular mechanisms involved in TM-mediated differentiation were therefore investigated. Reduced cell growth is known to be coupled to the differentiation process, and the cell cycle inhibitor p21^Cip1/WAF1^ is responsible for the early stages of the differentiation program^29^,^30^. The marker p21^Cip1/WAF1^ inhibits cell cycle progression by binding to proliferating cell nuclear antigen (PCNA) and blocking the ability of PCNA to activate DNA polymerase δ, the principal replicative DNA polymerase^31^,^32^. Therefore, levels of p21^Cip1/WAF1^ were determined and used as a differentiation index. The level of PCNA was also determined. As shown in Fig. 2A,B, stimulating THP-1 cells with PMA induced p21^Cip1/WAF1^ and inhibited PCNA expression relative to the untreated group (Fig. 2A,B, lane 2). To explore whether TM-associated signaling contributed to the effects of PMA treatment on p21^Cip1/WAF1^ expression, the cells were knocked down using TM siRNA prior to PMA treatment. The data showed that the level of p21^Cip1/WAF1^ was markedly decreased, and the level of PCNA was increased (Fig. 2A,B, lane 3). Conversely, the level of p21^Cip1/WAF1^ was increased when cells were transfected with HA-TM FL followed by PMA treatment (Fig. 2A, lane 4), suggesting that TM contributed to the effects of PMA-induced p21^Cip1/WAF^ expression in THP-1 cells. Furthermore, reduced cell growth/cell cycle arrest was also confirmed using flow cytometry, and treatment of cells with PMA for 5 days significantly induced cell cycle arrest. The percentage of cells in G0-G1 was 70.3 ± 2.9% compared to 19.6 ± 2.2.8% in the untreated control group. Transfecting cells with TM siRNA prior to PMA treatment significantly decreased the percentage of G0-G1 cells by 47.3 ± 3.7% relative to the PMA-treated group. The percentage of G0-G1 cells was enhanced to 84.1 ± 1.9% when THP-1 cells were transfected with HA-TM-FL followed by PMA treatment (Table 1), indicating that p21^Cip1/WAF^ expression, which was modulated by TM, promoted a reduction in cell growth in PMA-induced THP-1 cells. The mechanism by which TM regulates p21^Cip1/WAF1^ expression in PMA-stimulated THP-1 cells was then studied. The data demonstrated that exposing THP-1 cells to 150 nM PMA induced ERK1/2 phosphorylation (Fig. 2C, upper). Knockdown of TM using siRNA prior to PMA treatment inhibited ERK1/2 phosphorylation. The level of ERK1/2 phosphorylation was slightly increased when cells were transfected with HA-TM FL prior to PMA treatment (Fig. 2C, lower), suggesting that ERK1/2 phosphorylation, which is involved in PMA-induced THP-1 cell differentiation, is regulated by TM. NF-kB is one possible downstream signaling molecule of ERK1/2^33^. Therefore, western blot analysis for nuclear NF-kB p65 was also performed. Similar to ERK1/2 activation, nuclear NF-kB p65 is involved in PMA-induced THP-1 cell differentiation, which is regulated by TM (Fig. 2D). As shown in Fig. 2E, even though treatment of PD98059 alone for 72 hours did decrease the endogenous expression of TM in THP-1 cells, differentiation induced by PMA markedly increased p65 levels in the nucleus and p21^Cip1/WAF1^ expression, blockade of ERK1/2 using PD98059 abrogated the PMA-induced increase in p65 levels in the nucleus and p21^Cip1/WAF1^ expression, suggesting that TM-ERK1/2-NF-kB- and p65-mediated signaling played a crucial role in PMA-induced p21^Cip1/WAF1^ and PMA-decreased PCNA expression in THP-1 cells. In additions, as shown in Table 2, flow cytometry data indicated that the percentage of cells in G0-G1 significantly decreased to 40.9 ± 5.4% in the group pretreated with PD98059 followed by PMA stimulation relative to 70.3 ± 2.9% in the only PMA-treated group, confirming that ERK1/2 indeed played an important role in the PMA-induced reduction in cell growth. Finally, we analyzed the effects of PD98059 alone on THP-1 differentiation. It showed that treatment of PD98059 for 5 days did not change the rhythm of the cell cycle on THP-1 cells. The above results indicate that TM modulated p21^Cip1/WAF1^ expression via ERK1/2-NF-kB p65 signaling, which further promoted a reduction in cell growth, which is coupled to the differentiation process in PMA-induced THP-1 cells.

### TM interacted with and promoted the activation of cytoskeleton-associated molecules via ERK1/2 signaling during PMA-induced THP-1 cell differentiation

**Figure 3:**
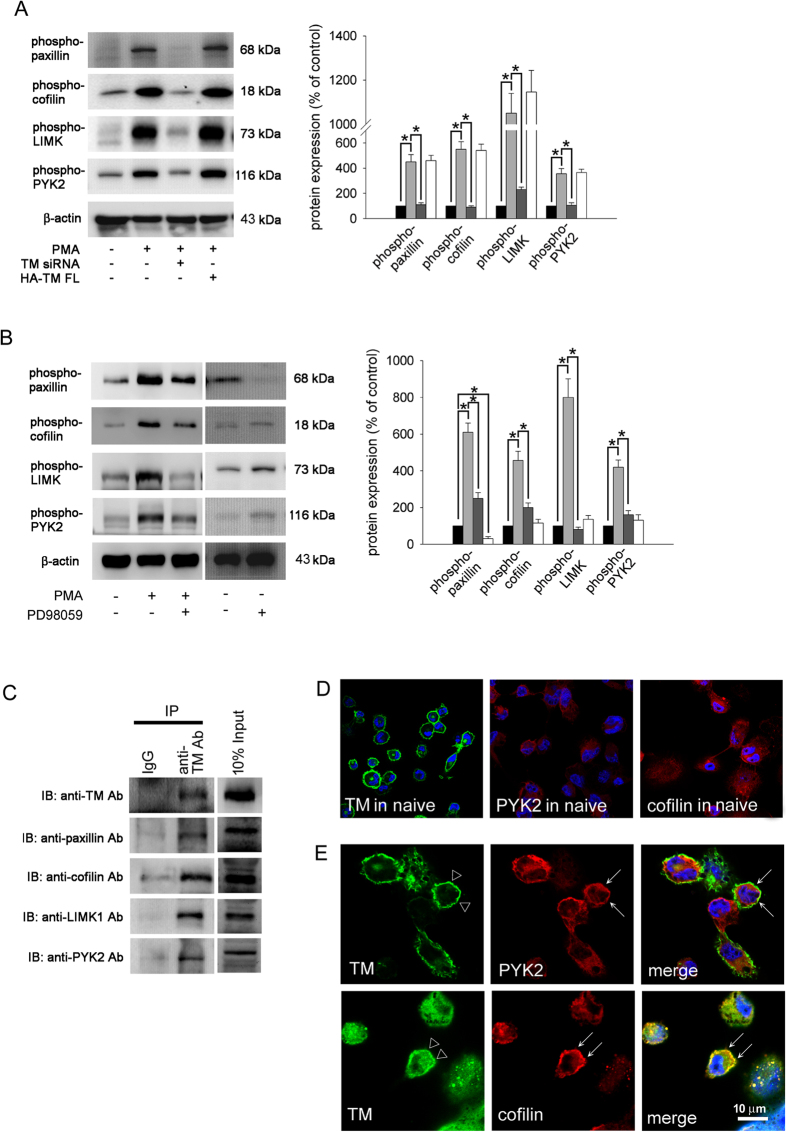
TM interacts with and activates cytoskeleton-associated molecules through ERK1/2 signaling in PMA-stimulated THP-1 cells. (**A**) THP-1 cells were transfected with TM siRNA (dark gray) or HA-TM FL plasmid (white) for 24 h followed by PMA stimulation for 72 hours (black: naïve THP-1 cells; light gray: PMA treatment). The total cell lysates were purified and analyzed for the activation (phosphorylation) of paxillin, cofilin, LIMK1, and PYK2 using western blot analysis. β-actin was used as a loading control. Five independent experiments have been performed (n = 5) for all studies, and representative images have been showed. The density of each band was quantified using densitometry and related protein expression was presented as bar graph. The data are presented as the mean ± SD, and *p < 0.05 was considered significant. (**B**) THP-1 cells were pretreated with 10 μM PD98059 (dark gray) for 1 h followed by PMA treatment for 72 hours (black: naïve THP-1 cells; light gray: PMA treatment; white: only PD98059 treatment). The total cell lysates were purified and analyzed for the activation (phosphorylation) of paxillin, cofilin, LIMK1, and PYK2 using western blot analysis. β-actin was used as a loading control. Five independent experiments have been performed (n = 5) for all studies, and representative images have been showed. The density of each band was quantified using densitometry and related protein expression was presented as bar graph. The data are presented as the mean ± SD, and *p < 0.05 was considered significant. (**C**) Lysates of THP-1 cells were immunoprecipitated using an anti-TM antibody or a rabbit IgG control followed by immunoblotting with antibodies against TM, paxillin, cofilin, LIMK1, or PYK2, as indicated. Five independent experiments have been performed for all studies (n = 5), and representative images have been showed. (**D**) Subcellular distributions of TM, PYK2 and cofillin in naïve THP-1 cells were detected by immunofluorescence and observed by confocal microscopy. (**E**) THP-1 cells were treated with PMA for 72 hours. Subcellular distributions of TM (triangle), PYK2 (arrow), and cofilin (arrow) in THP-1 cells were detected by immunofluorescence and observed by confocal microscopy. DAPI was used to stain the nuclei of THP-1 cells.

The cytoskeleton is the key component that regulates cell migration and differentiation^34^. To study the activation of cytoskeleton-associated molecules during THP-1 cell differentiation, western blot analysis for different phosphorylated forms of cytoskeleton-associated scaffolding proteins was performed. As shown in Fig. 3A, inducing differentiation with PMA markedly induced paxillin, cofilin, LIMK1, and PYK2 phosphorylation (Fig. 3A, lane 2) relative to levels in the untreated group. To determine whether TM contributes to these effects, TM expression was knocked down using TM siRNA prior to PMA treatment. The results showed that TM knockdown significantly inhibited paxillin, cofilin, LIMK1, and PYK2 phosphorylation (Fig. 3A, lane 3), suggesting that TM was also responsible for PMA-induced activation of cytoskeleton-associated molecules during THP-1 cell differentiation. Overexpression of TM followed by PMA treatment slightly increased the activation of these molecules relative to the only PMA-treated group (Fig. 3A, lane 4). To determine whether ERK1/2-mediated signaling also played roles in PMA-induced activation of cytoskeleton-associated molecules during THP-1 cell differentiation, THP-1 cells were pretreated with PD98059 followed by PMA stimulation. We found that the phosphorylation of paxillin, cofilin, LIMK1, and PYK2 was inhibited relative to the PMA-treated group (Fig. 3B, lane 3), suggesting that ERK1/2 signaling, which was responsible for PMA-induced p21^Cip1/WAF1^ expression, also played a crucial role in the activation of cytoskeleton-associated molecules during PMA-induced THP-1 cell differentiation. To determine whether TM interacts with these molecules in cells, immunoprecipitation (IP) assays were performed. As shown in Fig. 3C, IP-western analysis demonstrated that paxillin, cofilin, LIMK1, and PYK2 were present in the anti-TM antibody-immunoprecipitated fractions isolated from PMA-treated THP-1 cells (Fig. 3C lane 2), suggestion that TM can directly interact with cytoskeleton-associated molecules in PMA-stimulated THP-1 cells. In order to confirm the results of IP-western analysis, subcellular distributions of TM and cytoskeletons in THP-1 cells were detected by immunofluorescence and observed by confocal microscopy. Confocal microscopy showed that TM was predominantly found in the membrane of cells, and that naïve cells with dispersed distribution of PYK2 and cofillin (Fig. 3D). Interestingly, PMA may trigger a distribution change of cytoplasmic PYK2 and cofilin and make a colocalization with TM in THP-1 cells (Fig. 3E). These results suggested that TM directly interacts with and modulates paxillin, cofilin, LIMK1, and PYK2 phosphorylation via ERK1/2 signaling during PMA-induced THP-1 cell differentiation.

### PKCδ interacts with TM to regulate PMA-induced THP-1 cell differentiation

**Figure 4:**
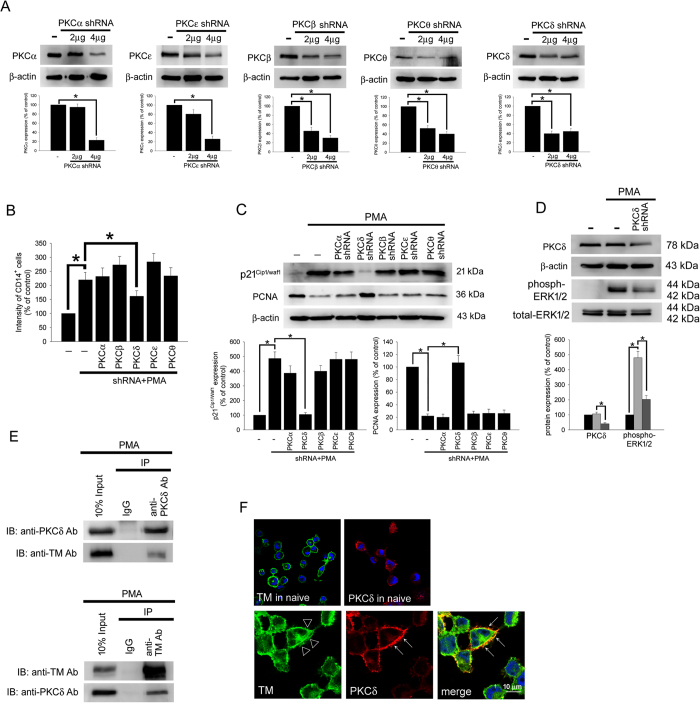
TM regulates THP-1 cell differentiation via the PKCδ-ERK1/2 signaling pathway. (**A**) THP-1 cells were transfected with 2 or 4 μg of PKCα, PKCβ, PKCδ, PKCε, or PKCθ shRNA for 24 h. Total cell lysates were purified, and knockdown efficiency was assayed using western blot analysis. (**B**) The THP-1 cells were knocked down by PKCα, PKCβ, PKCδ, PKCε, and PKCθ shRNAs for 24 h followed by PMA stimulation for 72 hours. The number of CD14^+^ cells was scored using flow cytometry. Data are expressed as a % of the control, are presented as the mean ± SD and represent the results of five independent experiments (n = 5, *p < 0.05 was considered significant). (**C**) Different sets of THP-1 cells were transfected with 4 μg of each shRNA for 24 h followed by PMA stimulation for 72 hours. The levels of p21^Cip1/WAF1^ and PCNA were analyzed using western blot analysis. (**D**) The THP-1 cells were knocked down by PKCδ shRNA for 24 h followed by PMA stimulation for 72 hours. The level of PKCδ and total and phosphorylated ERK1/2 was analyzed using western blot analysis. In western blot analysis, β-actin and total-ERK1/2 were used as loading controls. The density of each band was quantified using densitometry and related protein expression was presented as bar graph. The data are presented as the mean ± SD, and *p < 0.05 was considered significant (n = 5). (**E**) Lysates of THP-1 cells with PMA stimulation were extracted. Left, immunoprecipitated using goat anti-PKCδ or goat IgG control antibodies followed by western blot analysis and detected using goat anti-PKCδ or rabbit anti-TM antibodies. Right, immunoprecipitated using rabbit anti-TM or rabbit IgG control antibodies followed by western blot analysis and detected using rabbit anti-TM or goat anti-PKCδ antibodies. Five independent experiments have been performed (n = 5). (**F**) THP-1 cells were treated without (upper photos) or with (lower photos) PMA for 72 hours. Subcellular distributions of TM (triangle) and PKCδ (arrow) in THP-1 cells were detected by immunofluorescence and observed by confocal microscopy. DAPI was used to stain the nuclei of THP-1 cells.

In the above-described data, we show that TM and ERK signaling are associated with PMA-induced THP-1 cell differentiation. We identified a signaling pathway that links TM to ERK1/2 signaling and that induced both p21^Cip1/WAF1^ expression and the activation of cytoskeleton-associated proteins. However, the details of the molecular mechanisms that mediate TM’s function during ERK1/2 activation were not fully clear and were therefore explored. Increasing evidence has revealed that PMA treatment activates PKC and that it induces a greater degree of differentiation in THP-1 cells, which is reflected by increased adherence and the increased expression of surface markers that are associated with macrophage differentiation^13^. To determine the PKC isoform that is responsible for TM’s function in PMA-induced THP-1 cells, PKC isoforms, including PKCα, PKCβI, PKCδ, PKCε, and PKCθ, were knocked down using specific shRNAs (short-hairpin RNA) prior to PMA treatment, and the characteristics of macrophages were then assayed. First, we used western blot analysis to confirm the knockdown efficiency of each specific shRNA. The knockdown efficiency of these shRNAs in THP-1 cells (1 × 10^6^) that were showed in Fig. 4A. We found that knocking down the PKCδ isoform in THP-1 cells significantly decreased the CD14-positive cell labeling intensity that was induced by PMA treatment relative to the control PMA-treated group (Fig. 4B column 5). Furthermore, p21^Cip1/WAF1^ levels were markedly decreased and PCNA levels were increased in the group in which PKCδ was knocked down prior to PMA treatment (Fig. 4C lane 4), suggesting that PKCδ is the important PKC mediator during PMA-induced THP-1 differentiation. However, knockdown in THP-1 cells using the other PKCα, PKCβ, PKCε, and PKCθ shRNAs had no significant inhibitory effects. In addition, PKCδ knockdown strongly inhibited PMA-induced ERK1/2 activation (Fig. 4D lane 3), suggesting that PKCδ is the upstream molecule that mediates ERK1/2 activation in PMA-induced THP-1 cell differentiation. Schwende *et al*. reported that PMA-derived, but not VD3-derived, THP-1 differentiation resulted in the translocation of PKC isoenzymes to membrane fractions^13^. Hence, we hypothesized that TM may act as a scaffold for PKC docking. A co-IP assay was used to verify the interaction between TM and PKCδ in PMA-stimulated THP-1 cells. Western blot analysis showed that no signal was observed in the IgG IP control (Fig. 4E, left), whereas PKCδ was detectable in the anti-TM antibody immunoprecipitated fraction derived from PMA-stimulated THP-1 cells, indicating that there is an interaction between TM and PKCδ in THP-1 cells. In order to further confirm the interaction between TM and PKCδ, immunoprecipitation using goat anti-PKCδ antibody was also done. The data indicated that the co-immunoprecipitated TM was obviously detected in anti-PKCδ antibody immunoprecipitate (Fig. 4E). Additionally, confocal microscopy showed that the PKCδ was predominantly and dispersedly found in the naïve THP-1 cells. PMA may trigger the activation of PKCδ, and make PKCδ colocalized with TM in THP-1 cells (Fig. 4F). These results indicate that PKCδ, which interacts with TM, is the molecule that links TM to ERK1/2 signaling. We therefore suggest that TM may act as a scaffold for PKCδ docking, which could keep PKCδ in the region close to the membrane and activate downstream signaling.

### ERK1/2 regulates p21^Cip1/WAF1^ expression and PKCδ interacts with TM in human PBMC

**Figure 5:**
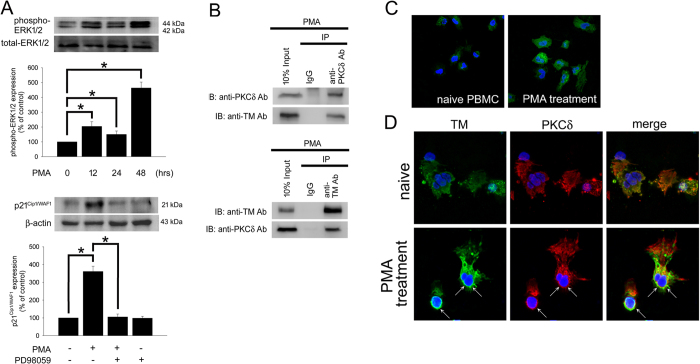
ERK1/2 regulates p21^Cip1/WAF1^ expression and PKCδ interacts with TM in PMA-stimulated PBMCs, which is consistent with those results in THP-1 cells. (**A**) Human PBMCs were stimulated with 150 nM PMA for 12–48 h. Upper, the expression of phosphorylated ERK1/2 in total cell lysates were assayed using western blot analysis. Lower, human PBMCs were treated with 150 nM PMA, pretreated with 10 μM PD98059 for 1 h followed by PMA treatment, or treated with PD98059 alone for 48 h. A western blot assay was performed to determine the expression of p21^Cip1/WAF1^. The total-ERK1/2 and β-actin were used as loading controls. Three independent experiments (PBMCs from 3 voluntary donors) have been performed (n = 3), and representative images have been showed. The density of each band was quantified using densitometry and related protein expression was presented as bar graph. The data are presented as the mean ± SD, and *p < 0.05 was considered significant. (**B**) Lysates of PBMCs with 150 nM PMA stimulation for 24 hours were extracted. Upper, immunoprecipitated using goat anti-PKCδ or goat IgG control antibodies followed by western blot analysis and detected using goat anti-PKCδ or rabbit anti-TM antibodies. Lower, immunoprecipitated using rabbit anti-TM or rabbit IgG control antibodies followed by western blot analysis and detected using rabbit anti-TM or goat anti-PKCδ antibodies. (**C**) PBMCs were treated with 150 nM PMA for 24 hours. The expression of CD68 in PBMCs was detected by immunofluorescence and observed by confocal microscopy. (**D**) PBMCs were treated without (upper photos) or with (lower photos) PMA for 24 hours. Subcellular distributions of TM (arrow) and PKCδ (arrow) in PBMCs were detected by immunofluorescence and observed by confocal microscopy. DAPI was used to stain the nuclei of PBMCs. Three independent experiments (PBMCs from 3 voluntary donors) have been performed, and representative images were presented.

In order to confirm those phenomena which were observed in THP-1 cells, the selected confirmatory experiments were performed using human peripheral blood mononuclear cells (PBMCs). The western blotting results demonstrated that exposing PBMCs to 150 nM PMA for 12–48 hours induced ERK1/2 phosphorylation (Fig. 5A, upper). Additionally, p21^Cip1/WAF1^ expression by PMA markedly increased, blockade of ERK1/2 using PD98059 abrogated the PMA-induced increase in p21^Cip1/WAF1^ levels (Fig. 5A, lower), and suggesting that ERK1/2 -mediated signaling played a critical role in PMA-induced p21^Cip1/WAF1^ expression in human PBMCs. A co-IP assay was used to verify the interaction between TM and PKCδ in PMA-stimulated PBMCs. Western blot analysis showed that no signal was observed in the IgG IP control (Fig. 5B, upper), whereas PKCδ was detectable in the anti-TM antibody immunoprecipitated fraction derived from PMA-stimulated PBMCs, indicating that there is an interaction between TM and PKCδ in PBMCs. In order to further confirm the interaction between TM and PKCδ, immunoprecipitation using goat anti-PKCδ antibody was also done. The data indicated that the co-immunoprecipitated TM was obviously detected in anti-PKCδ antibody immunoprecipitate (Fig. 5B, lower). Additionally, confocal microscopy showed that the PMA treatment may trigger the expression of CD68 in PBMCs (Fig. 5C). PKCδ was predominantly and dispersedly found in the naïve PBMCs. PMA may trigger the activation of PKCδ, and make PKCδ colocalized with TM in PBMCs (Fig. 5D). These results indicate that ERK1/2 activation regulates p21^Cip1/WAF1^ expression and PKCδ interacts with TM in PMA-stimulated PBMCs, which is consistent with those results in THP-1 cells.

### PKCδ is highly expressed in human atherosclerotic arteries and co-localizes with TM in infiltrated macrophages

**Figure 6:**
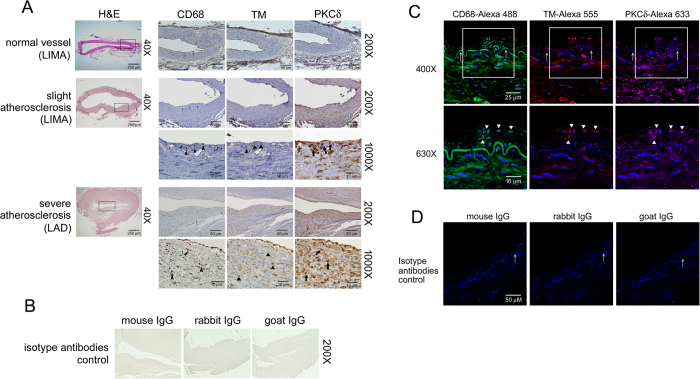
The expression of TM and PKCδ in infiltrated macrophages in human atherosclerotic arteries. (**A**) Immunohistochemistry was used to assay the expression of CD68, TM and PKCδ in human LIMA and LAD. The expression of CD68, TM, and PKCδ was determined using specific antibodies, and tissues were counterstained with hematoxylin. CD68 antibodies were used to identify the infiltrated macrophages in vessel walls. H&E staining showed that the intima was thickened in both slightly atherosclerotic LIMA and severely atherosclerotic LAD (black rectangle). Images at 200X and 1000X have been shown. The black arrows indicate the internal elastic lamina. In addition, CD68, TM, and PKCδ^+^ cells are indicated with black arrowheads, triangles, and thick arrows, respectively. Six human samples (3 for slight atherosclerosis and 3 for severe atherosclerosis) have been analyzed and representative images have been showed. (**B**) The mouse isotype IgG, rabbit isotype IgG, or goat isotype IgG were used as controls. (**C**) The expression of CD68, TM and PKCδ in human atherosclerotic vessels were identified using immunofluorescent triple staining. DAPI was used to stain the nuclei of cells. Photos are shown the same regions as 400X magnifications of tissues on slides in the upper column. The white arrows indicate the internal elastic lamina. In addition, images at 630X are shown (white rectangles on 400X slides), and CD68, TM, and PKCδ^+^ cells are indicated with black triangles. (**D**) The mouse isotype IgG, rabbit isotype IgG, or goat isotype IgG were used as controls for immunofluoresent staining.

To investigate the pathophysiological significance and *in situ* expression of TM and to confirm the evidence shown in *in vitro* studies, atherosclerotic arteries were evaluated using immunohistochemistry. Clinical specimens from patients who underwent CABG (coronary artery bypass grafting) surgery and heart transplantation were obtained. The arrows in the Fig. 5 indicated the internal elastic lamina. There did not have thickened intima or atherosclerotic lesion formation in the normal vessel. In contrast, markedly clarified neointima in both slightly and severely atherosclerotic arteries were observed. Staining with anti-CD68 Ab for identification of infiltrated macrophages showed that less macrophages infiltrated into the vessel walls in the control vessel compare to atherosclerotic vessels. The expression of TM was predominant on endothelium in normal vessel. In contrast, significant inhibition of TM on endothelium was observed in atherosclerotic vessel. The atherosclerotic vessels contain higher levels of PKCδ expression and macrophage infiltration. Furthermore, high power magnification (1000X) revealed strong TM (triangle) expression in neointima-infiltrated CD68-postive macrophages (arrowhead). Strong PKCδ (thick arrow) staining was also detectable (Fig. 6A). We also got the similar presentation in immunofluorescent triple staining (Fig. 6C). These results indicate that PKCδ is highly expressed in human atherosclerotic arteries and that it co-localizes with TM in infiltrated macrophages, suggesting that TM and PKCδ coordinate in macrophages to participate in atherogenesis.

## Discussion

This report describes the direct evidence that suggests an important role for endogenous TM in PMA-induced THP-1 cell differentiation, demonstrating that the signaling molecules that mediate macrophage differentiation are regulated by TM. TM is a cell surface-expressed transmembrane glycoprotein. It consists of 557 amino acids, and it contains 5 domains, including a highly charged N-terminal lectin-like domain (D1), a domain with six epidermal growth factor (EGF)-like structures (D2), a serine and threonine-rich domain (D3), a transmembrane domain (D4) and a cytoplasmic domain (D5)^17^. Recent evidence has revealed that TM, especially its lectin-like domain, performs potent anti-inflammatory functions independent of its anticoagulant activity, demonstrating a potential therapeutic role for recombinant TM protein for the treatment of inflammatory diseases^35^. In addition, Cheng *et al*. have reported that TM is implicated in keratinocyte differentiation and wound healing. Primary cultured keratinocytes obtained from TM^lox/lox^; K5-Cre mice (keratinocyte-specific TM deletion mice) underwent abnormal differentiation in response to calcium stimulation. Downregulation of loricrin and filaggrin indicated poor epidermal differentiation and wound healing were observed in TM^lox/lox^; K5-Cre mice. Silencing TM expression in human epithelial cells impaired calcium-induced extracellular signal-regulated kinase pathway activation and subsequent keratinocyte differentiation. In addition, local administration of recombinant TM (rTM) improved healing rates in the skin of TM-null mice. Hence they suggested that TM plays a critical role in skin differentiation and wound healing^36^. Previous studies also focused on the ability of TM to mediate anti-inflammatory effects via its lectin-like domain. The TM lectin-like domain might regulate inflammation by maintaining the integrity of cell-cell interactions and preventing the transmigration of leukocytes^37^. TM maintains epithelial morphology and promotes cell migration by the direct interaction of its cytoplasmic domain and ezrin which associates with actin filaments^38^. Additionally, our *in vitro* findings suggested that TM interacts with the actin cytoskeleton and simultaneously interacts with intersectin, further supported a role for TM in cell migration^27^. The cytoskeleton is a key component that allows cells to maintain proliferation, migration, and differentiation. LIMK1 activates actin depolymerizing factor/cofilin and induces actin reorganization. The phosphorylation of PYK2 and paxillin associate with the focal adhesion complexes formation and cell migration in monocytes^39^,^40^. Thus, we predict that TM may interact with ezrin, intersectin, and actin filaments, which result in regulation of cell differentiation.

However, the expressions of TM in various monocytic cell lines are differences. As early as 1993, Kizaki K *et al*. had evidenced that TM mRNA levels were induced by 0.1–10 nM of PMA in monocytic, macrophagic and neutrophilic cells differentiated from HL-60 cells^41^. Except U937, THP-1 and HL-60 cells expressed high levels of surface TM. Stimulation with LPS or TNF-α up-regulated TM expression by mononuclear phagocytes^42^. Our previous study demonstrated that TM is expressed in monocytes and that its intracellular domain acts as a negative regulator that inhibits cytoskeleton activation in IL-6-mediated THP-1 cell migration. TM siRNA increases IL-6-induced THP-1 cell chemotaxis and migration by activating ERK1/2 and JNK/SAPK signaling^27^. However, in this study, we showed that TM siRNA decreases PMA-induced THP-1 cell differentiation via the inactivation of ERK1/2 and NK-κB signaling, indicating a diversity of roles for TM in monocyte functions that depend on the stimuli received by the cell. In 2012, Yang *et al*.^28^ demonstrated that recombinant human soluble TM (rTM) induced THP-1 cell growth arrest and differentiation via the activation of JNK/c-Jun signaling. In addition, they found that 1,25-(OH)_2_D3 treatment enhanced rTM-induced cell growth arrest and differentiation. They also transfected THP-1 cells with TM plasmid and found that the forced expression of TM increased the percentage of THP-1 cells that expressed CD14, which resulted in the up-regulation of p-JNK, VDR, and C/EBPα in these cells, supporting the idea that TM is a positive regulator of monocytic leukemia cell differentiation. Although recombinant human soluble TM is suggested to be a potent stimulator of monocyte differentiation, the roles of endogenous TM in PMA-induced THP-1 differentiation and its regulatory mechanisms are not yet fully understood. Our pilot study found that TM expression was increased soon after PMA induction; thus, we focused on the roles and underlying mechanisms by which TM regulates these cell processes. We used gain-of-function (HA-TM FL transfection) and loss-of-function (TM siRNA) approaches to define the involvement of TM in PMA-induced THP-1 cell differentiation, and we suggest that TM may act as a scaffold for a direct interaction with PKCδ, which contributes to subsequent ERK1/2 signaling activation and results in THP-1 cell differentiation. Our advanced data (not demonstrated in this text) evidenced that domain 5 (intracellular domain) of TM is involved in PMA-dependent paxillin, cofilin, and LIMK1 signaling pathway that regulate cell differentiation. We are manufacturing the serial deleted constructs to identify the minimal region required for TM interaction with PKCδ; using the site-directed mutagenesis technique to provide evidence of the requirement for the peptides in this interaction. If the binding domain of TM may be measured, it could represent a promising approach to prevent inflammation and atherosclerosis in patients via the regulation of TM expression.

During the process of lesion formation and vascular inflammation in atherosclerosis, macrophage differentiation, proliferation, cell growth arrest, and apoptosis *et al*. all are essential for pathogenesis^43^,^44^,^45^. In early stage of atherosclerosis, macrophages limit lesion progression by phagocytizing oxidized lipoproteins, dead cells, and cellular debris. Unfortunately, the macrophages in advanced lesions contribute to an inappropriate inflammation that can lead to irreversible plaque formation^45^. However, in this complex and gradual process, many potential factors are involved, such as p21^Cip1/WAF1^, PCNA, G1 cyclin-CDK *et al*. Reduced cell growth is coupled to differentiation. Additionally, the cell cycle inhibitor p21^Cip1/WAF1^ is responsible for the early stages of the differentiation program^28^,^29^. p21^Cip1/WAF1^ inhibits cell cycle progression by binding to G1 cyclin-CDK complexes via its N‐terminal domain. In addition, p21^Cip1/WAF1^ also binds to PCNA at its C‐terminal domain and thereby blocks the ability of PCNA to activate DNA polymerase δ, which is the principal replicative DNA polymerase^32^,^46^. Therefore, combined with the previous evidences and our finding in this study, we predict that TM-PKCδ may inhibit cell growth in monocytes. Indeed, we have been studying this important issue *in vitro* study and in cardiac surgery patients.

The transient activation of ERK1/ERK2 is sufficient to induce the expression of p21^Cip1/WAF1^ in G1 phase^47^. Our study demonstrated that HA-TM-FL transfection significantly enhanced PMA-induced cell growth arrest by increasing the level of p21^Cip1/WAF1^ through ERK1/2-NF-kB p65 signaling, which increased the percentage of cells in G0-G1stage cell and promoted differentiation. The PKC isoforms α, β, and δ are implicated in the induction of macrophage differentiation in normal progenitor cells as well as in human and murine myeloid cell lines^48^,^49^. M-CSF induced the rapid catalytic activation of PKC-δ and the membrane translocation of phosphorylated PKC-δ in myeloid precursor cells^50^. Schwende *et al*. demonstrated that PMA caused a strong increase in PKCδ and a weak increase in PKC-α, PKC-ε, and PKC-ξ expression^13^. The studies described above strongly suggest that PKCδ may be the key molecule that is involved in the TM-associated differentiation of THP-1 cells that is induced by PMA. Consistent with this finding, our data showed that knockdown of the PKCδ isoform in THP-1 cells significantly decreased CD14-positive cell labeling intensity, ERK1/2 activation, and p21^Cip1/WAF1^ levels, suggesting that PKCδ is the important PKC mediator that is the upstream molecule that mediates ERK1/2 activation in PMA-induced THP-1 cell differentiation.

Macrophages participate in intracellular lipid accumulation and foam cell formation in early atherosclerosis lesions^5^. However, uptake of low-density lipoprotein (LDL) by macrophages via scavenger receptors is one of the critical steps in atherogenesis^51^. Cholesterol loading of macrophages stimulates the production of inflammatory mediators, such as cytokines and reactive oxygen species, that recruit other cell types and contribute to the development of a complex lesion^52^. The studies described above strongly indicate that macrophage differentiation is an essential process during atherosclerosis development. Therefore, determining the mechanisms by which monocytes differentiate into macrophages is critical to understanding how to prevent atherosclerosis. In the presence of agonists of RXR (retinoid X receptor), a member of the nuclear hormone receptor superfamily, PMA-induced THP-1 cell differentiation was inhibited, suggesting that RXR and its agonists may be useful for therapeutic applications in the future through these anti-atherosclerotic effects^53^. Similar to our finding, in 1997, Oida *et al*. demonstrated that PMA also increased the expression level of TM in THP-1 cells^54^. However, they also found that incubation with oxLDL resulted in an increase of TM levels in THP-1 cells, but did not induce cells differentiation to the macrophages. Although up-regulation of TM in PMA-stimulated THP-1 cells had been demonstrated, here we hypothesized that TM is a key regulator of PMA-induced THP-1 cell differentiation. We further studied TM’s regulating mechanisms in detail, which may be helpful for understanding the atherosclerotic effect of endogenous TM on monocyte differentiation and for the development of therapeutic applications in the further.

**Figure 7:**
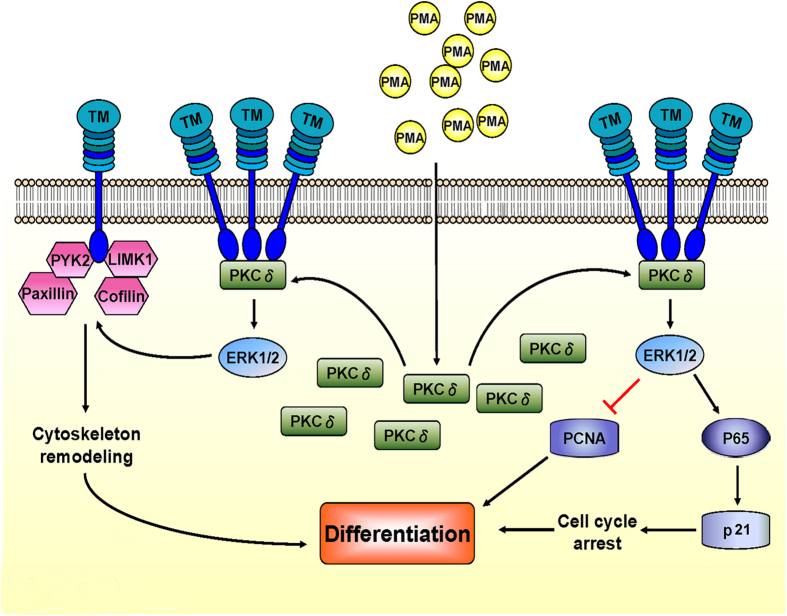
Mechanism by which TM contributes to PMA-mediated differentiation in THP-1 cells. TM expression is increased soon after PMA induction in THP-1 cells. TM may regulate PMA-induced THP-1 cell differentiation via a direct interaction with PKCδ. TM acts as a scaffold for PKCδ docking, which keeps PKCδ in the region close to the membrane and promotes subsequent ERK1/2 activation. ERK1/2 activation subsequently enhances the expression of cell cycle inhibitor p21^Cip1/WAF1^ via NF-kB p65 signaling, which causes cell cycle arrest and promotes differentiation. On the other hand, ERK1/2 activation participates in the phosphorylation of paxillin, cofilin, LIMK1, and PYK2, which interact with TM and mediate cytoskeleton remodeling to promote differentiation.

In conclusion, the schematic diagram shown in Fig. 7 summarizes the mechanism by which TM contributes to the PMA-mediated differentiation of THP-1 cells. TM expression is first increased when THP-1 cells are exposed to PMA. TM then acts as a scaffold for PKCδ docking, which keeps PKCδ in the region close to the membrane and promotes subsequent ERK1/2 activation. On the one hand, ERK1/2 activation enhances the expression of the cell cycle inhibitor p21^Cip1/WAF1^ via NF-kB p65 signaling, which causes cell cycle arrest and promotes differentiation. ERK1/2 activation participates in the phosphorylation of paxillin, cofilin, LIMK1, and PYK2, which interact with TM to mediate cytoskeletal remodeling to promote differentiation. The data strongly suggests that the TM expression participates in PMA-induced macrophage differentiation, and provides a novel view of the role of TM in macrophage differentiation during the development of atherosclerosis.

## Methods

### Cell culture and differentiation

A human pro-myelomonocytic cell line, THP-1 cells, were obtained from the American Type Culture Collection (ATCC, VA, USA) and grown in RPMI 1640 medium (Thermo-Fisher Scientific Inc, CA, USA) with 2 mM L-glutamate, 4.5 g/L glucose, 10 mmol/L HEPES, 1.0 mmol/L sodium pyruvate, 10% fetal bovine serum, and 1% antibiotic-antimycotic mixture. Cell density was maintained between 5 × 10^4^ and 8 × 10^5^ viable cells/mL, and the medium was refreshed every 48–72 hours. The cells were differentiated into a macrophage-like phenotype by incubating them with 150 nM PMA in complete medium for 72 hours at 37 °C in 5% CO_2_.

### Extraction and treatment of human peripheral blood mononuclear cells

Human total PBMCs were isolated from 50 ml peripheral blood of healthy young human volunteers by density gradient centrifugation with Histopaq-1077 (density 1.077 g/ml; Sigma, MO, USA) according to the user instrument. Extracted PBMNCs were plated in RPMI 1640 medium with supplements (2 mM L-glutamate, 4.5 g/L glucose, 10 mmol/L HEPES, 1.0 mmol/L sodium pyruvate, 10% fetal bovine serum, and 1% antibiotic-antimycotic mixture) on cultured plates at 37 °C in a 5% CO_2_ incubator for 2 hours, then treated cells with 150 nM PMA and performed subsequent studies.

### Knockdown of TM gene expression by siRNA transfection

The knockdown of TM gene expression was achieved by siRNA transfection. THP-1 cells (1 × 10^6^) were suspended in 2.5 mL of serum-free medium and transfected with 25 μM of TM siRNA duplexes (Invitrogen Catalog THBD-HSS110719, 110721, 186320, NY, USA) for 6 hours using Lipofectamine® RNAiMAX (Invitrogen, Carlsbad, CA, USA) according to the manufacturer’s instructions. Then, the cells were resuspended in complete medium and analyzed for siRNA knockdown efficiency 24 h after transfection using flow cytometry and western blot analysis.

### Knockdown of PKC gene expression by shRNA electroporation

shRNAs against human PKCα, PKCβII, PKCδ, PKCε, and PKCθ were purchased from National RNAi Core Facility User Committee Academia Sinica, Taipei, Taiwan. THP-1 cells were transfected with shRNAs via electroporation using the Neon transfection system (Invitrogen, Carlsbad, CA, USA) according to the manufacturer’s instructions. A total of 1 × 10^6^ cells were transfected with 2 or 4 μg of each shRNA plasmid and then cultured with complete medium without antibiotic-antimycotic mixture at 37 °C in 5% CO_2_. The cells were analyzed for shRNA knockdown efficiency 24 h after electroporation using western blot analysis.

### Construction and overexpression of HA-tagged TM full-length expression vectors in electroporated THP-1 cells

The pCDNA3.1/V5-His-TM-FL plasmid, which contains a segment of the TM open reading frame, was a gift from Professor Yu-Jia Chang, Taipei medical University, Taiwan. For the construction of the influenza hemagglutinin (HA) epitope-tagged full-length TM expression plasmid (HA-TM FL), the pCDNA3.1/V5-His-TM plasmid was first digested with the SacI and XhoI enzymes, filled in with Klenow, and subsequently cloned in-frame into the SmaI sites of the pXJN-HA vector, which contains the chick β-globin intron downstream (3′) of the CMV promoter. THP-1 cells were transfected with HA-TM FL plasmids via electroporation using the Neon transfection system (Invitrogen, Carlsbad, CA, USA) according to the manufacturer’s instructions. A total of 1 × 10^6^ cells were transfected with 2 μg of HA-TM FL plasmid. The cells were analyzed for overexpression 24 h after electroporation.

### Macrophage identification by Liu and immunofluorescence staining

The PMA-treated THP-1 cells or PBMCs (1 × 10^5^) were rinsed with PBS, fixed in 4% paraformaldehyde, and spun onto glass slides using a Shandon Cytospin 4 Cytocentrifuge (Thermo Scientific, Pittsburgh, PA, USA). The macrophages were first identified using Liu staining and examined using an Axio Imager A1 Microscope (Carl Zeiss Micro Imaging Inc., Thornwood, NY, USA). The slides were stained using either PE-conjugated mouse anti-human CD14 (25–0149, eBioscience, San Diego, CA, USA) or FITC-conjugated mouse anti- human CD68 (11–0689, eBioscience), and nuclei were identified using Hoechst 33258 (Sigma, St. Louis, MO, USA). Images were obtained using an Axio Imager A1 microscope (Carl Zeiss Micro Imaging Inc., Thornwood, NY, USA).

### Flow cytometry for macrophage cell surface marker analysis

The identification and characterization of THP-1 differentiation were also performed using flow cytometry. THP-1 cells were harvested and incubated with PE-conjugated mouse anti-human CD14 (25–0149, eBioscience), FITC -conjugated mouse anti-human CD68 (11–0689, eBioscience), or a mouse IgM/IgG2a isotype control (DakoCytomation, Produktionsvej, Glostrup, Denmark) for 60 min. After washing the cells with buffer (phosphate-buffered saline containing 0.1% bovine serum albumin and 0.1% sodium azide), the expression of the cell surface markers CD14 and CD68 was analyzed using flow cytometry on a FACSCalibur™ (BD Biosciences, Franklin Lakes, New Jersey, USA).

### Cell cycle analysis by DNA content using flow cytometry

A total of 2 × 10^6^ THP-1 cells were fixed with 70% ethanol at 4 °C for at least 24 hours. Cells were washed three times with cold PBS and resuspended in PI staining solution containing 25 μg/ml PI, 40 μg/ml RNAse, and 0.3% Tween-20 at room temperature for 30 minutes. Finally, cell DNA contents were analyzed using flow cytometry on a FACSCalibur™ (BD Biosciences, Franklin Lakes, New Jersey, USA).

### Western blot analysis

We placed 3 × 10^6^ cells in 10 cm dish for western blot analysis. We collected the suspended and attached THP-1 cells, and the total cell lysates were extracted. Protein concentrations were determined using a Bio-Rad Protein Assay Kit (BioRad Inc., CA, USA), with BSA used as the standard. For each blot, approximately 50 μg of total protein was fractionated by SDS-PAGE and transferred to a PVDF (polyvinylidene difluoride) membrane. The membranes were respectively probed with rabbit anti-human TM (TA307719, OriGene Technologies, Inc, Rockville, MD, USA), rabbit anti- p21^Cip1/WAF1^ (#2947, Cell Signaling, Danvers, CA, USA), rabbit anti-PCNA (sc-7907, Santa Cruz biotechnology, Santa Cruz, CA, USA), mouse anti-phospho-p44/42 MAPK (Erk1/2) (Thr202/Tyr204) (#9106, Cell Signaling, Danvers, CA, USA), rabbit anti-p44/42 MAPK (Erk1/2)(#9102, Cell Signaling, Danvers, CA, USA), rabbit anti-phospho-NF-κB p65 (Ser536)(#3033, Cell Signaling, Danvers, CA, USA), rabbit anti-Paxillin (phospho Y31)(ab32115, Abcam, MA, USA), rabbit anti-Paxillin (ab32084), rabbit anti-Cofilin (phospho S3)(ab47281, Abcam, MA, USA), rabbit anti-Cofilin (ab54532, Abcam, MA, USA), rabbit anti-LIM Kinase 1 (phospho T508)(ab38508, Abcam, MA, USA), rabbit anti-LIM Kinase 1 (ab87971, Abcam, MA, USA), rabbit anti-PYK2 (phospho Y881)(ab4801, Abcam, MA, USA), rabbit anti-PYK2 (ab32571, Abcam, MA, USA), mouse anti-PKC α (sc-8393, Santa Cruz Biotechnology, Santa Cruz, CA, USA), rabbit anti-PKC βII (sc-210, Santa Cruz Biotechnology, Santa Cruz, CA, USA), goat anti-PKC θ (sc-1875, Santa Cruz Biotechnology, Santa Cruz, CA, USA), goat anti- PKCδ (sc-937-G, Santa Cruz Biotechnology, Santa Cruz, CA, USA), and rabbit anti-PKC ε (sc-214, Santa Cruz Biotechnology, Santa Cruz, CA, USA) antibodies. The membranes were then incubated with horseradish peroxidase (HRP)-conjugated goat anti-rabbit IgG antibodies (Merck Millipore, Darmstadt, Germany), HRP-conjugated goat anti-mouse IgG antibodies (Merck Millipore, Darmstadt, Germany) or HRP-conjugated donkey anti-goat IgG antibodies (Amersham, Arlington Heights, IL, USA). The proteins were visualized using an enhanced chemiluminescence (ECL) detection kit (Amersham Biosciences, NJ, USA) and scanned by Odyssey Imaging System (LI-COR, Inc., Lincoln, NE, USA).

### Immunoprecipitation

THP-1 cells transfected with either TM siRNA or PKCs shRNA and untransfected controls were lysed in buffer containing 150 mM NaCl, 50 mM Tris–HCl (pH 7.4), 1% NP-40, and 1X protease inhibitor cocktail (Sigma, MO, USA). Lysed cells were placed on ice for 30 minutes and then centrifuged at 10,00 × g for 30 minutes to remove cell debris. Approximately 500 μg of protein extract was pre-cleaned with 20 μL of a 50% protein A/G suspension mixture (protein A: BioRad, Inc., CA; protein G: Amersham, IL, USA). Pre-cleaned lysates were immunoreacted with rabit anti-human TM antibody (TA307719, OriGene Technologies, Inc, Rockville, MD, USA) or rabbit IgG (Sigma, St. Louis, MO, USA) at 4 °C for 16 h and then immunoprecipitated by adding 50 μL of a 50% protein A/G sepharose mixture at 4 °C for 1.5 hrs. The beads were washed three times with ice-cold lysis buffer, and the bound proteins were eluted with 2X SDS–PAGE loading buffer and subjected to western blot analysis.

### Human artery collection and immunohistochemical/immunofluorescent staining

Human left internal mammary arteries (LIMA) were obtained from patients who underwent coronary artery bypass graft (CABG) surgery, and left coronary arteries (LAD) were obtained from the patients who underwent heart transplantation. The Ethics Committee of Tri-Service General Hospital approved this study (TSGHIRB 100-05-232), which conformed to the requirements of the Declaration of Helsinki, and all patients gave informed consent prior to all procedures. Immunohistochemical staining was performed on serial and 5 μm-thick paraffin-embedded cross-sections of LIMA and LAD using mouse anti-human CD68 (sc-20060, Santa Cruz biotechnology, Santa Cruz, CA, USA), rabbit anti-human TM, (TA307719, OriGene Technologies, Inc, Rockville, MD, USA), and goat anti- human PKCδ (sc-937-G, Santa Cruz biotechnology, Santa Cruz, CA, USA) antibodies. The anti-mouse, rabbit, and goat IgG peroxidase-conjugated or Alexa Fluo-conjugated secondary antibodies were purchased from Amersham (Arlington Heights, IL, USA) or Invitrogen (Carlsbad, CA, USA). The nuclei were counterstained with hematoxylin or DAPI. The mouse IgG, rabbit IgG, or goat IgG isotype antibodies were use as controls. One of the serial sections was assayed using hematoxylin and eosin (H&E) to determine the vessel morphology. Images were acquired using Axio Imager A1 microscope (Carl Zeiss Micro Imaging Inc., Thornwood, NY, USA).

### Statistical analyses

In this experiment, we performed each test for five times (n = 5), including *in vitro* cell experiments. The amount of protein expression in western blot analysis was quantified using ImageQuant TL software (version 7.0; GE Healthcare Life Sciences, Marlborough, MA, USA). The β-actin, total-ERK1/2, or lamin B1 were used for normal loading controls/normalization. The ratio of the abundance of the target protein to the normal loading control is used to quantify the amount of the target protein in each sample. The expression level of target protein was presented as % of control group or fold of control group. Statistical analysis was performed using the SigmaStat software (version 3.5; SPSS, Chicago, IL, USA). Between groups analysis of summary statistic was performed using Student’s t-tests and one- or two-way ANOVA followed by Dunnett’s test. Value was expressed as the means ± SD and statistical significant was set at P < 0.05.

## Additional Information

**How to cite this article**: Tsai, C.-S. *et al*. Thrombomodulin regulates monocye differentiation *via* PKCδ and ERK1/2 pathway *in vitro* and in atherosclerotic artery. *Sci. Rep.* **6**, 38421; doi: 10.1038/srep38421 (2016).

**Publisher's note:** Springer Nature remains neutral with regard to jurisdictional claims in published maps and institutional affiliations.

## Figures and Tables

**Table 1:** The DNA content of THP-1 cells in PMA administration for 5 days.

	control	TM si RNA	HA-TM FL	PMA administration for 5 days		
				—	TM si RNA	HA-TM FL
G_0_/G_1_ phase	19.6 ± 2.8%	20.5 ± 3.2%	18.7 ± 3.1%	70.3 ± 2.9%*	47.3 ± 3.7%†	84.1 ± 1.9%*†
S phase	58.9 ± 6.2%	54.2 ± 5.0%	53.4 ± 7.0%	8.9 ± 3.1%*	29.7 ± 5.2%†	11.2 ± 3.5%*
G_2_/M phase	16.2 ± 8.8%	25.3 ± 9.1%	27.9 ± 3.4%	10.6 ± 0.8%*	6.2 ± 1.0%*	4.5 ± 1.9%*

(n = 5). *p < 0.05 compared to control group; ^†^p < 0.05 compared to PMA-administrated group.

**Table 2:** The effects of ERK1/2 inhibitors in PMA-administrated THP-1 cells.

	control	PD98059	PMA administration for 5 days	
			—	PD98059
G_0_/G_1_ phase	19.6 ± 2.8%	20.5 ± 3.1%	70.3 ± 2.9%*	40.9 ± 5.4%*^†^
S phase	58.9 ± 6.2%	60.1 ± 7.5%	8.9 ± 3.1%*	15.9 ± 2.8%*^†^
G_2_/M phase	16.2 ± 8.8%	15.7 ± 6.8%	10.6 ± 0.8%*	43.2 ± 5.1%*

(n = 5). PD98059, ERK1/2 inhibitor. *p < 0.05 compared to control group; ^†^p < 0.05 compared to PMA-administrated group.
